# Targeting the Sphingolipid Rheostat in Gliomas

**DOI:** 10.3390/ijms23169255

**Published:** 2022-08-17

**Authors:** Faris Zaibaq, Tyrone Dowdy, Mioara Larion

**Affiliations:** Neuro-Oncology Branch, National Cancer Institute, National Institutes of Health, Bethesda, MD 20814, USA

**Keywords:** sphingolipids, ceramide, sphingosine-1-phosphate, rheostat, brain tumors, isocitrate dehydrogenase 1 mutation, gliomas

## Abstract

Gliomas are highly aggressive cancer types that are in urgent need of novel drugs and targeted therapies. Treatment protocols have not improved in over a decade, and glioma patient survival remains among the worst of all cancer types. As a result, cancer metabolism research has served as an innovative approach to identifying novel glioma targets and improving our understanding of brain tumors. Recent research has uncovered a unique metabolic vulnerability in the sphingolipid pathways of gliomas that possess the IDH1 mutation. Sphingolipids are a family of lipid signaling molecules that play a variety of second messenger functions in cellular regulation. The two primary metabolites, sphingosine-1-phosphate (S1P) and ceramide, maintain a rheostat balance and play opposing roles in cell survival and proliferation. Altering the rheostat such that the pro-apoptotic signaling of the ceramides outweighs the pro-survival S1P signaling in glioma cells diminishes the hallmarks of cancer and enhances tumor cell death. Throughout this review, we discuss the sphingolipid pathway and identify the enzymes that can be most effectively targeted to alter the sphingolipid rheostat and enhance apoptosis in gliomas. We discuss each pathway’s steps based on their site of occurrence in the organelles and postulate novel targets that can effectively exploit this vulnerability.

## 1. Introduction

Gliomas are aggressive and highly invasive neoplasms, with one of the poorest patient outcomes of all cancer types. They are the most common type of intracranial cancer and comprise more than 80% of all malignant brain tumors [1]. The severity and prevalence of gliomas have made them a key focus of cancer biology research, resulting in discoveries such as the chemotherapeutic temozolomide (TMZ). The standard of care for brain tumors was last updated in 2005 when TMZ was formally approved for use in several types of gliomas and glioblastomas [2]. Initially, this was heralded as a significant change in the way we approach and treat brain tumors, and it has indeed been a lifesaver for many patients [3]. Despite this progress however, the median survival rate of patients suffering from glioblastoma (GBM), the most common and severe type of glioma [1], remains well under one year [3]. These stagnant survival statistics demonstrate the urgent need for continued research that aims to develop more effective therapies.

In recent years, this has prompted the exploration of novel approaches to targeting brain tumors, which has resulted in an increased interest in glioma metabolism [4]. One of the most significant metabolic markers of gliomas is a mutation in the isocitrate dehydrogenase (IDH*^mut^*) enzyme of the tricarboxylic acid cycle (TCA) [5,6,7]. This early-onset mutation [8] is common in lower-grade gliomas and causes gliomagenesis [9] through the excess production of D-2-hydroxyglutarate (D-2HG) [10]. The presence of the IDH mutation in gliomas strongly impacts the molecular etiology of the disease and significantly affects patient survival [6]. These differences between IDH*^mut^* and IDH*^wt^* tumors prompted the World Health Organization to reconsider its brain tumor classification system to include the IDH status of gliomas [6].

Our group recently discovered a metabolic vulnerability within IDH*^mut^* glioma subtypes that may contribute to improved patient survival [11]. These tumors present with unique sphingolipid metabolism that is directly induced by their mutant IDH status and sensitizes them to the inhibition of sphingosine kinase 1 [11]. This disrupts the production of the oncopotent sphingosine-1-phosphate and alters the balance of sphingolipids to induce apoptosis in glioma cells [11]. Based on this research, we concluded that the sphingolipid pathway offers potential targets for translating novel therapies from the lab bench to the clinic to improve outcomes in glioma patients [11]. These findings directed us to explore current translational research and identify other sphingolipid pathway enzymes that can be targeted to exploit this vulnerability in gliomas and enhance apoptosis.

**Figure 1:**
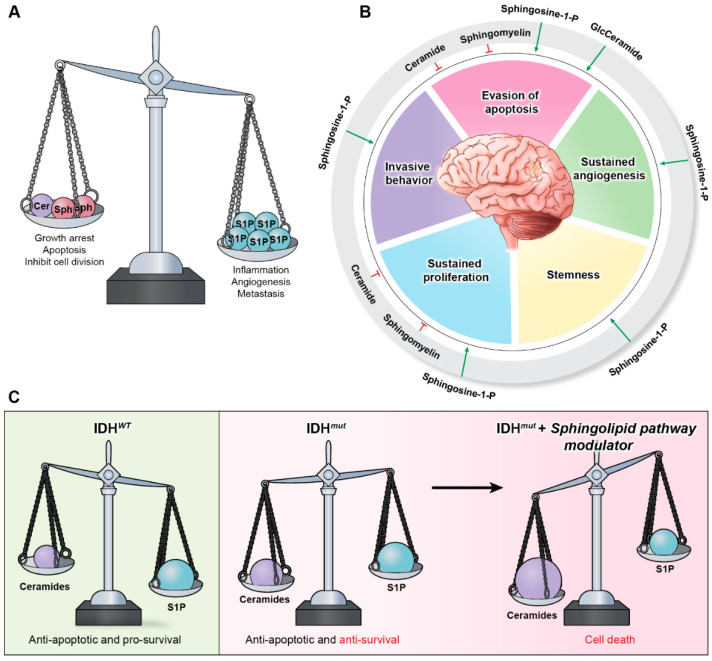
The interplay between ceramide and S1P forms the basis of the sphingolipid rheostat and is the focus of great interest because of its implications for the hallmarks of cancer. (**A**) Sphingolipid rheostat and how this balance can affect cell growth. (**B**) Key effects of significant sphingolipids on the hallmarks of cancer. (**C**) Vulnerabilities of the rheostat of IDH*^mut^* tumors make them susceptible to drugs that induce ceramide accumulation or S1P depletion. Therefore, sphingolipid pathway modulators may play a key role in altering the rheostat and diminishing the hallmarks of cancer.

Sphingolipids are a family of lipid signaling molecules that play a variety of second messenger functions in cellular regulation. The two primary metabolites, sphingosine-1-phosphate (S1P) and ceramide, maintain rheostat balance and play opposing roles in cell survival and proliferation [11]. S1P is a potent promoter of sustained proliferation, while various ceramide species are known for inducing apoptosis [11,12]. The ratio of these two signaling molecules is known as the sphingolipid rheostat (Figure 1A), which has profound implications for several cellular processes, including many hallmarks of cancer (Figure 1B). S1P’s signaling activities allow it to promote angiogenesis [13], invasiveness [14], stemness [15], proliferation [16], and evasion of apoptosis [17] in gliomas [11,18], as well as many other cancer types. Meanwhile, ceramide signaling inhibits cell division and induces cell death [11]. It comes as no surprise that glioblastoma (GBM) cells have altered sphingolipid homeostasis, typically decreasing ceramides and upregulating S1P production to promote their survival [11,13,15,19].

However, our recent discovery revealed that IDH*^mut^* gliomas possess an imbalanced sphingolipid rheostat that results in elevated ceramide and sphingosine production relative to IDH*^wt^* gliomas (Figure 1C) [11]. This vulnerability makes IDH*^mut^* gliomas more susceptible to agents that can alter the rheostat, causing a shift from S1P production toward ceramide accumulation [11]. In addition, it provides a likely explanation for the characteristic tendency of IDH*^mut^* tumors to be lower in grade and less aggressive than their IDH*^wt^* counterparts. Recently, these discoveries have generated interest in targeting the sphingolipid pathway enzymes that can lead to increased ceramide production or suspended S1P production [11,20,21]. Altering the rheostat such that the pro-apoptotic signaling of the ceramides outweighs the pro-survival signaling of S1P in glioma cells diminishes the hallmarks of cancer and enhances tumor cell death [11].

The rheostat can be altered by targeting enzymes that directly affect the ceramides-to-S1P balance, or by targeting other pathway enzymes that can indirectly induce ceramide accumulation or S1P depletion. Additionally, certain classes of chemotherapeutic drugs are known to increase endogenous ceramide levels in cancers [22,23,24]. These studies indicate that administering a maintenance therapy that alters the rheostat alongside chemotherapy could encourage ceramide production and enhance tumor death [25,26,27,28,29,30]. By identifying which enzymes are the most suitable targets, this review intends to help to pave the way for more focused and efficient sphingolipid therapy development.

While malignancy in glioma subtypes has been positively correlated with the expression of sphingosine kinase and subsequent S1P production, translational research investigating targets along the sphingolipid pathway in gliomas is limited and remains quite novel within the neuro-oncology community [11]. The established understanding of the sphingolipid pathway, as well as the role of S1P and other sphingolipids, is mainly based on non-glioma cancers. Throughout this review, we analyze the pathway to identify which enzymes can be most effectively targeted to alter the sphingolipid rheostat and enhance apoptosis in gliomas. This is conducted in reference to the specific glioma subtypes IDH1*^mut^* or IDH1*^wt^*, which possess characteristically distinct sphingolipid metabolism [11]. Additionally, we discuss each enzyme’s potential as a sphingolipid target in the context of its organelle localization to more accurately relate enzyme biochemistry to glioma biology. We focus on published works that describe the sphingolipid pathway in detail and explore the translational aspect of targeting this pathway in gliomas.

## 2. Sphingolipid Pathway

Although these discoveries will be discussed in greater depth in the following sections, it is important to provide a brief overview of the basic mechanisms that ultimately control the rheostat balance between S1P and ceramide. The synthesis and fate of ceramides is an essential theme of sphingolipid metabolism, which is mediated by the activity of a series of enzymes from interconnected pathways: de novo ceramide synthesis, the sphingomyelin pathway, and the ceramide salvage pathway [31]. Consequently, the movement of metabolites through the sphingolipid pathway is discussed in the context of these groups of enzymes in this review.

Serine palmitoyltransferase (SPT) is the rate-limiting step of the de novo ceramide synthesis portion of the sphingolipid pathway, which condenses serine and palmitoyl-CoA to form 3-ketosphinganine [32]. 3-Ketosphinganine reductase (KDSR) then catalyzes the NADPH-dependent formation of sphinganine (dihydrosphingosine). Dihydroceramide synthase (CerS) acylates sphinganine with specificity for acyl-CoA donors to form dihydroceramides. Dihydroceramide is then irreversibly reduced by dihydroceramide desaturase (DEGS) to form ceramide. Alternatively, CerS also participates in the salvage pathway and can convert sphingosine to ceramide.

Upon its synthesis in the endoplasmic reticulum (ER), ceramide can be shunted to the Golgi body either via the ceramide transfer protein (CERT) [32] or through vesicular trafficking. Its mechanism of entering the Golgi body determines its potential fate, as each pathway attributes ceramide to different synthetic enzymes that determine its role in cell signaling and regulation. Sphingomyelin synthase catalyzes the conversion of phosphatidylcholine and ceramide to release 1,2-diacylglycerol (DAG) and sphingomyelin (SM), which is later hydrolyzed by sphingomyelinases (SMPD) to release ceramide. Ceramide kinase (CerK) phosphorylates ceramide, which is later dephosphorylated by ceramide 1-phosphate phosphatase. In addition, ceramide can also enter glycosphingolipid synthesis (e.g., glucocerebrosides, glycodihydroceramides, lactosylceramide, sulfatides) following glycosylation by glucosylceramide synthase (UGCG). Ceramide is later released following hydrolysis by glucocerebrosidase.

While ceramide has numerous potential fates, the hydrolysis of ceramide to form sphingosine by ceramidase (ASAH1) heavily influences the sphingolipid rheostat. The subsequent accumulation of sphingosine results in anti-proliferative signaling in a manner that resembles ceramide-induced apoptosis [31]. The successive phosphorylation of sphingosine via sphingosine kinase (particularly SPHK1) produces sphingosine-1-phosphate (S1P), which is the primary pro-survival sphingolipid. Sphingosine-1-phosphate phosphatase (SGPP) catalyzes the reverse reaction by dephosphorylating S1P to release sphingosine, while sphingosine-1-phosphate lyase (SGPL) degrades long-chain bases as they exit the pathway. Ceramide and S1P maintain a negative correlation (via the sphingolipid-rheostat balance), in which the upregulation of ceramide is directly correlated with the concomitant downregulation of S1P, and vice versa [31].

## 3. Palmitic Acid Uptake and Incorporation into the Sphingolipid Pathway

Palmitic acid (PA) is one of two metabolites required to initiate sphingolipid biosynthesis, and its metabolism before entering the pathway can dictate its function and destination in the cell. In addition to sphingolipid metabolism, PA is a ubiquitous fatty acid in human cells that plays a critical role in health and disease by modulating membrane homeostasis, energy storage, and protein palmitoylation [33]. As a result, many cancers, including gliomas [34], selectively upregulate PA synthesis via de novo lipogenesis [35], which facilitates the increased S1P synthesis observed in gliomas [11]. Due to its importance, cells possess complex mechanisms to regulate the uptake, cytoplasmic concentration, and metabolic roles of PA. In this section, we discuss the journey of PA as it enters the brain and highlights the key enzymes that facilitate its entry into the sphingolipid pathway.

### 3.1. PA Transport into the Brain

Early in vivo lipid studies of rat brains indicate that radiolabeled PA crosses the blood-brain barrier (BBB) and is readily incorporated into every major class of brain lipids, including sphingomyelin and complex ceramides [36]. Since PA is a saturated fatty acid (FA) with a 16-carbon backbone, its diffusion across the BBB does not occur fast enough to meet physiological demands [37]. As a result, PA diffusion is supplemented by several proteins known to transport FA. An mRNA analysis of human brain microvascular endothelial cells identified FATP1 (fatty acid transport protein), FAT/CD36 (fatty acid translocase), and FABP5 (fatty acid-binding protein) as the most common central nervous system (CNS) transporters, with varying PA affinities [37,38].

**Figure 2:**
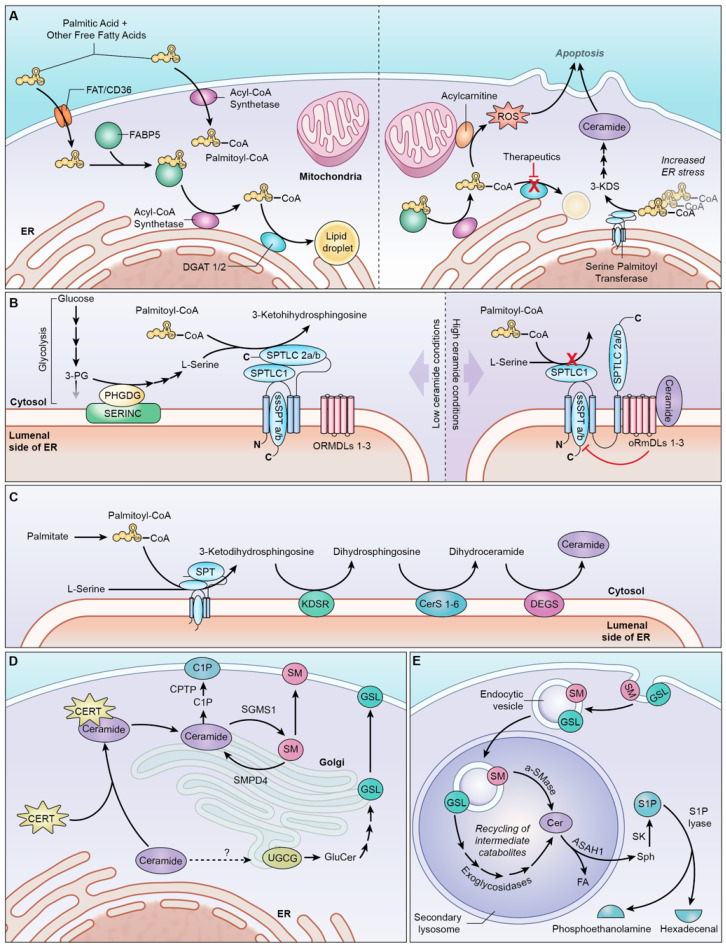
Sphingolipid synthesis and biology. (**A**) FA import and storage in lipid droplets. Interruption of this process can result in FA accumulation in the cytosol, inducing ER stress, ROS production, and eventually apoptosis. (**B**) SPT complex, demonstrating the roles of different subunits. ORMDLs are known to inhibit catalytic activity in high-ceramide conditions. (**C**) De novo ceramide synthesis occurs via four consecutive enzymatic steps in the cytoplasmic leaflet of the ER membrane. (**D**) De novo ceramide can be transported to the Golgi for sphingomyelin, ceramide-1-p, or glycosphingolipid synthesis. These metabolites are then transported to the cell membrane, where they play various signaling roles related to the hallmarks of cancer. (**E**) The cell endocytosis parts of the cell membrane, which merges with enzyme-containing lysosomes to degrade sphingolipids.

Of these PA transporters, FAT/CD36 and FABP5 are highly correlated with decreased glioma patient survival, and both are expressed at significantly higher levels in IDH*^wt^* tumors [39]. As seen in Figure 2A, FAT/CD36 is a stationary integral membrane protein that allows a wide range of FA to cross the cell membrane. By contrast, FABP5 facilitates the movement of C16–18 FA (e.g., PA) across the cell membrane and towards intracellular destinations within the cell, such as the ER, where it can enter the sphingolipid pathway. An extensive metabolic investigation is warranted to examine the roles of each PA transporter in different glioma subtypes. Specific inhibitors targeting FABP5 and FAT/CD36 have been developed and successfully tested in other disease models, which can be applied to glioma research [40,41].

### 3.2. PA Storage in Lipid Droplets

Upon entering the glioma cell, PA can be dedicated to various pathways, including beta-oxidation, membrane lipid production, or sphingolipid biosynthesis. However, excess PA accumulation induces lipotoxicity in T98G [42] and U87 GBM cells [43] by triggering the formation of ROS and promoting the ER stress pathways [42]. This can ultimately lead to apoptosis at PA concentrations greater than 300 μM [42,43]. IDH*^wt^* glioma cells neutralize the lipotoxicity of PA and other free fatty acids by incorporating them into triacylglycerols (TAGs) and storing them in lipid droplets (LD) [43]. This reaction is catalyzed by the two isoforms of diacylglycerol acyltransferase (DGAT), whose activity quickly enhances the effects of PA lipotoxicity [42].

The inhibition of DGAT1 has proven especially effective in preventing TAG synthesis in various patient-derived IDH*^wt^* glioblastoma cell lines [43,44]. It minimizes the uptake and storage of PA in LDs and diminishes the cytoprotective properties of the DGAT enzymes [44]. As a result, the lipotoxicity escape mechanism of glioma cells is hindered, resulting in cytoplasmic PA accumulation rather than sequestration in LDs. In this scenario, the excess PA is shunted towards other cellular pathways [45], such as sphingolipid metabolism [46], contributing preferentially to ceramide synthesis [44,46] due to the existing lipotoxicity-related ER stress (Figure 2A) [47]. The inhibition of DGAT1 has proven especially effective in preventing TAG synthesis in various patient-derived IDH*^wt^* glioblastoma cell lines [43,44]. The knockdown of DGAT1 increases glioma ceramide levels in vitro [44], and DGAT1 inhibition with the drug T863 reduces the volume of LDs in the GBM cells [43]. Additionally, treating IDH*^wt^* glioblastoma xenograft mouse models with another DGAT1 inhibitor, A-922500, induces apoptosis and significantly reduces the tumor size [44]. The clear correlation between DGAT1 inhibition and sphingolipid production in gliomas makes this a promising target for continued therapeutic investigation.

There has also been a significant discovery revealing a potential metabolic role of the DGAT2 enzyme in cancer [48]. DGAT2 mediates its putative effects by binding with other sphingolipid enzymes and producing 1-O-acylceramide from cytosolic ceramides, which is promptly sequestered in LDs [49]. Its potential to affect the sphingolipid rheostat is discussed in greater depth in Section 3.3 and Section 4.2.

### 3.3. PA Activation for Sphingolipid Synthesis

To participate in de novo sphingolipid biosynthesis, PA must first be activated by an acyl-CoA synthetase (ACS). A total of 26 enzymes in the body possess ACS activity [50], and they are categorized based on their specificity in varying the FA chain lengths. In the ER, ACS enzymes play three important roles in sphingolipid metabolism. ACS enzymes activate (1) the PA required to initiate de novo synthesis, (2) other FA chain residues designated for dihydroceramide formation, and (3) degradation products that exit the pathway [51]. Each ACS has a unique substrate specificity, localization, and tissue distribution that defines its function. Therefore, each ACS can funnel different types of FA towards distinct cellular and metabolic fates [50,52,53]. Due to the close homology of many ACS enzymes, specific inhibitors and modulators have not yet been developed to discretely target individual isoforms. To that end, we examine the metabolic roles of five ACS isoforms that potently manipulate the glioma rheostat to propose novel targets.

Both acyl-CoA synthetase long-chain (ACSL)1 and ACSL5 possess an affinity for PA that exceeds that of the other ACS isoforms [54,55]. Although ACSL5 is not common in healthy brain tissue, it is highly expressed in glioma cells [56] and contributes to enhanced glioma survival under acidotic conditions [57]. The oncogenic properties of ACSL5 are dependent on its catalytic activity [56,57], leading some researchers to hypothesize that ACSL5 is responsible for activating PA for sphingolipid synthesis [49]. However, Senkal et al. revealed that ACSL5 participates in a novel pathway that neutralizes ceramides to prevent the induction of apoptosis. Both ACSL5 and ACSL1 interact with DGAT2 (discussed in Section 3.2) and ceramide synthase (discussed in Section 4.2) at the ER/lipid droplet interface in liver cells to produce acylceramides, which are promptly sequestered in lipid droplets [49]. The interruption of acylceramide synthesis by inhibiting ACSL5 or DGAT2 results in ceramide accumulation, which enhances the caspase activation [49]. Meanwhile, cells that overexpress ACSL5 or DGAT2 exhibit lower ceramide levels [49]. Therefore, ACSL1 and ACSL5 appear to play significant roles in altering ceramide levels in a cell. Given that no tissue-specific isoforms have been reported for these enzymes, the ACSL/CerS/DGAT interaction may occur in other tissues, such as the brain.

In support of this hypothesis, Zhou et al. note that ACSL1 and ACSL5 expression is significantly higher in IDH*^wt^* gliomas compared to IDH*^mut^* [58]. Their pro-tumorigenic presence in IDH*^wt^* gliomas could protect the tumor by preventing increased ceramide levels. Meanwhile, IDH*^mut^* gliomas lack this protection and are more susceptible to apoptosis [11]. Despite their potential as targets in gliomas, no preclinical studies have developed inhibitors to selectively target ACSL1 or ACSL5. The development of selective ACSL inhibitors could lead to novel therapies for glioma patients and improve our understanding of sphingolipid metabolism.

ACSL6 is one of two isoforms that are dominant in nervous tissue [59], while ACSL4 is more moderately expressed [60]. These isoforms’ activation of long-chain PUFA results in the production of pro-apoptotic ceramides [11]. Additionally, elevated enzyme expression in gliomas results in dramatically improved patient survival [39]. Patient-derived glioma samples present with a lower expression of both ACSL4 [44] and ACSL6 [39] compared to normal tissue, likely to mitigate the production of pro-apoptotic ceramides.

A metabolomic study using patient-derived glioma xenografts supports this potential role of both isoforms in the sphingolipid rheostat of IDH*^mut^* tumors [58]. Levels of activated PUFA were notably higher in IDH*^mut^* cell lines compared to IDH*^wt^* [58,61], which is likely due to the increased expression of ACSL4 and ACSL6 [39,58] in the former. Therefore, ACSL4 and 6 may contribute to the increased C18–24 ceramide levels observed in IDH*^mut^* gliomas, contributing to their metabolic vulnerabilities [11]. Additionally, a recent study has directly linked the enzymatic activity of ACSL4 to the induction of ferroptosis and the subsequent suppression of the proliferation of several glioma cell lines [62]. Evaluating drugs that activate or increase the expressions of these ACS enzymes, particularly ACSL6, because of its preferential expression in the CNS, could help to target IDH*^mut^* glioma metabolism in ways that positively impact patient outcomes.

Acyl-CoA synthetase very long-chain (ACSVL)3 is the only ACS enzyme whose activity has been explicitly tied to aberrant sphingolipid metabolism and increased glioma proliferation [63]. This recent development means that this enzyme is of particular interest to our review. Despite its primary preference for C18-C22 FA [63] and non-expression in the healthy brain [63], ACSVL3 is substantially expressed in malignant IDH*^wt^* gliomas (e.g., U87 and Mayo 22 cells) [64]. Zhou et al. also found that ACSVL3 was elevated in more aggressive IDH*^wt^* tumors [58], which resulted in lower patient survival [39]. Its differential expression between IDH*^wt^* and IDH*^mut^* gliomas may be another contributing factor to the unique sphingolipid rheostat and enhanced vulnerability of the IDH*^mut^* subtypes [11].

ACSVL3 knockout in U87MG IDH*^wt^* glioblastoma cells results in dramatic reductions in C18-C22 ceramide and S1P levels [63], thus altering the two most important components of the sphingolipid rheostat. While cell growth can be significantly reduced by ACSVL3 knockdown, cell survival is not impacted, resulting in continued (albeit decreased) proliferation [64]. In U87 IDH*^wt^* glioblastoma xenograft mouse models, ACSVL3 knockdown resulted in a tumor growth rate decreased by 60% compared to the controls [64]. Given this data, an ACSVL3-specific inhibitor could play a promising role as a maintenance therapy that enhances the effectiveness of chemotherapeutics.

## 4. Endoplasmic Reticulum: De Novo Synthesis of Ceramide

Upon the dedication of PA to sphingolipid synthesis, palmitoyl-CoA is ready to undergo condensation with L-serine, which is the first step of the sphingolipid pathway. This reaction is catalyzed by serine palmitoyltransferase (SPT), a large and closely regulated enzyme complex that initiates the de novo synthesis of ceramide. De novo ceramide production involves a series of four consecutive enzymatic steps that all take place in the cytosolic leaflet of the endoplasmic reticulum (ER) (Figure 2C) [65]. Although none of the four enzymes form a complex with one another [66], the similar localizations and consecutive roles of SPT [67], KDSR [68], CerS [69], and DEGS [70] contribute to efficient metabolic flux through the pathway. This results in pools of ceramide produced by the ER, which can perpetuate its metabolism in the Golgi or signal for apoptosis.

The inhibition of enzymes in the de novo pathway reduces the total sphingolipid synthesis throughout the cell. However, altering the absolute quantity of the sphingolipids does not necessarily impact the sphingolipid rheostat and tip the scales towards either ceramide or S1P production. Since the rheostat ratio is paramount to glioma cell survival, it is crucial to identify targets that can specifically shift the rheostat towards the pro-apoptotic sphingolipids. Serine palmitoyltransferase and dihydroceramide desaturase have been the focus of therapeutic development. Meanwhile, the close homology of ceramide synthase enzymes has hindered the development of specific inhibitors that can target individual isoforms, similar to the ACS enzymes. KDSR also has minimal effects on the cell and no known inhibitors, limiting its potential as a therapeutic target.

### 4.1. First Step of Sphingolipid Synthesis

Serine palmitoyltransferase (SPT) is a large complex composed of catalytic and regulatory subunits that modulate the first step of sphingolipid biosynthesis, each of which can be uniquely targeted. In addition to palmitoyl-CoA, the second metabolite required for sphingolipid production is L-serine [71]. Consequently, astrocytes and other supporting glial cells synthesize L-serine through glycolytic intermediates to ensure an adequate supply throughout the CNS [71,72].

L-serine molecules are assigned to sphingolipid synthesis by the SERINC (serine incorporator) scaffold proteins, which co-localize with SPT in the ER membrane [72]. SERINCs are expressed throughout the rat brain and act as a scaffold for 3-phosphoglycerate dehydrogenase [72], the cytoplasmic enzyme that assigns the glycolytic intermediates to serine synthesis. The close clustering of serine synthetic enzymes with SPT likely creates a pool of L-serine dedicated to sphingolipid metabolism (Figure 2B). SERINC1 is particularly notable because its overexpression in the brain results in enhanced SPT activity and sphingolipid production [72]. Further research may elucidate the unique impacts of each of the five SERINC isoforms on sphingolipid synthesis and help to develop inhibitors that interfere with the protein interactions.

The catalytic portion of SPT is a heterodimer comprised of the hLCB1 (SPTLC1) subunit, forming either of two potential dimers with hLCB2a (SPTLC2) or hLCB2b (SPTLC3) [73]. These two primary subunits catalyze the condensation of L-serine and palmitoyl-CoA to form 3-ketodihydrosphingosine (3-KDS), the main precursor of the long-chain bases (LCBs) of sphingolipids. The discovery and investigation of natural sphingolipid analogs (typically found in fungi) have led to an interest in the specific inhibition of SPT. The inhibition of the catalytic subunits in U87MG IDH*^wt^* glioblastoma cells induced a 50% reduction in the cell count and resulted in statistically significant reductions in the S1P concentrations [74].

A dimer comprising two primary (catalytic) subunits alone generates minimal SPT activity. To adequately produce sphingolipids, one of the two “small subunits” (ssSPTa or ssSPTb) must be associated with a heterodimer [73], resulting in four possible subunit combinations. Of these combinations, ssSPTa binding with the hLCB1/2a dimer creates the most active SPT isoform, which produces the most standard long-chain bases (LCBs) [67]. Elevated ssSPTa expression increases catalytic activity by nearly 100-fold [73]. Therefore, endogenous levels of ssSPTa are the limiting factor for the SPT catalytic activity [73], which is the rate-limiting step of the sphingolipid biosynthesis [75]. Pharmacological interventions that target ssSPTs provide a promising direction for novel therapies.

SPT catalytic activity is also closely regulated by the inhibitory orosomucoid-like proteins (ORMDLs) [76]. There are three constitutively expressed and functionally redundant ORMDL isoforms [77] that form a stable complex with the multi-subunit SPT [78]. As shown in Figure 2B, the ORMDLs can inhibit sphingolipid biosynthesis in response to ceramide accumulation [77,79]. This tight regulation of ceramide production comes as no surprise, given the ceramides’ pro-apoptotic nature, along with the importance of balancing the sphingolipid rheostat.

The ORMDL-induced inhibition of SPT is likely mediated by de novo ceramide in the ER [77,80], which is sensed by a hitherto unidentified ceramide-binding domain on the ORMDLs [79]. This allosteric feedback induces a conformational change in the ORMDL structure, shifting the proteins into the “inhibitory” state that significantly reduces the catalytic activity of the SPT [79]. The simultaneous knockdown of all three ORMDLs is necessary to prevent the inhibition of SPT activity [77]. Although the ORMDLs may be phenotypically redundant [77], their mechanisms of action may differ upon interaction with different ceramides or varying concentrations of ceramide [79]. This negative feedback system plays an important role in regulating sphingolipid production, and further investigation is necessary to clarify its mechanism in gliomas to target it pharmacologically.

### 4.2. Production of Dihydroceramide

Due to the limited impacts of KDSR, the next enzyme of significance is ceramide synthase (CerS). The six CerS isoforms rapidly condense the sphinganine produced by KDSR with a fatty acyl-CoA of varying chain lengths and saturations to produce a dihydroceramide [81]. CerS isoforms also participate in the salvage pathway by utilizing sphingosine as a substrate to form ceramides [82]. Additionally, each CerS isoform possesses distinct tissue distributions and FA specificities [83], implying that each produces a unique set of (dihydro)ceramides that play differing physiological roles in the body.

CerS3 is the only CerS isoform to possess specificity for alpha hydroxy FA and PUFA up to 32 carbons long, resulting in unique (dihydro)ceramide products [84]. Some of these non-standard hydroxy-ceramides induce pro-apoptotic signaling in C6 glioma cells [85]. Additionally, CerS3 expression is higher in IDH*^mut^* tumors, which correlates with improved patient outcomes [39]. Given that IDH*^mut^* gliomas are uniquely susceptible to imbalances in their sphingolipid rheostat [11], CerS3 activity may contribute to the production of non-standard ceramide species (within the lipidome analysis) that promote apoptosis [11].

The next two isoforms, CerS1 and CerS4, are both significantly expressed in the brain [83]. Both CerS1 [81] and CerS4 [86] localize to the ER and readily metabolize either sphinganine or sphingosine as their long-chain base substrate [86,87]. Therefore, both isoforms are primed to participate in downstream sphingolipid production. Since these two isoforms are the most abundant in the CNS and possess high specificity for C18 acyl-CoA [69,81], the pro-apoptotic C18 ceramide [88] is the most common ceramide species in the brain [69,83]. The induced overexpression of CerS1 in the IDH*^wt^* U251 and A172 glioma cells results in increased C18 ceramide levels and induces autophagy [88]. To maintain a pro-survival rheostat, certain IDH*^wt^* gliomas possess C18 ceramide levels that are significantly lower than those found in healthy brain tissue [13,88].

In the acylceramide pathway involving the DGAT/ACSL/CerS complex (detailed above), Cers1 and CerS4 possess the greatest molecular affinity for ACSL5 compared to all other CerS isoforms [49]. This may have important implications for the sphingolipid rheostat, given that the pro-apoptotic C18 ceramide produced by Cers1 and CerS4 [88] is preferentially sequestered to lipid droplets in certain gliomas. This acylceramide pathway, proposed by Senkal et al., may serve as a regulatory mechanism to reduce the cytosolic levels of C18 ceramide in certain non-glioma cell lines [49]. Further studies must be conducted to validate this proposed pathway in the brain, which could lead to novel treatments.

While CerS2 presents with a minimal expression throughout the CNS, it produces an over-representative portion of the ceramide and sphingomyelin species, as well as the vast majority of hexosylceramides in the brain [83]. CNS glycosylceramides almost exclusively contain a C22, C24, or C24:1 fatty acyl moiety [83], which are the primary fatty acyl substrates for CerS2 [69]. This occurrence is likely due to the specificity of the ceramide transport (CERT) protein, which translocates ceramides from the ER to the Golgi (discussed further in Section 5.1). While the (dihydro)ceramides produced by CerS2 are typically pro-apoptotic [11], they are directed towards the production of pro-survival [89] glycosphingolipids in the brain [83,90]. Therefore, the upregulation of glycosphingolipid synthesis serves as a metabolic sink and removes ceramides from the cytosolic pool. Uniquely, CerS2 possesses a non-competitive S1P binding site, which decreases enzyme activity when S1P concentrations are elevated [83]. This unique feature of CerS2 can be exploited through the development of novel drugs that are specific to this allosteric binding site, which would exclusively target CerS2.

CerS5 and CerS6 are robust producers of C16 ceramide, which plays a critical role in mammalian cells [69]. It has been described as having both pro- and anti-apoptotic properties in various cell lines and physiologic conditions. Interestingly, recent metabolomic studies have found that C16 ceramide levels in gliomas are equal to or higher than the levels observed in healthy tissue, particularly in aggressive GBM lines [13,88,91]. Moreover, it has been reported that C16 ceramide serves as a growth promoter in certain tumors, particularly heterogenous IDH*^mut^* gliomas [11]. Additional research is required to determine whether targeting CerS5 and CerS6 elicits beneficial biostatic responses in glioma subtypes.

### 4.3. Production of Ceramide

During the final step of de novo ceramide synthesis (Figure 2C), dihydroceramide desaturase (DEGS) catalyzes the irreversible addition of a 4–5 trans double bond to the sphinganine backbone to produce a ceramide [92]. This critical step is responsible for the functionality and signaling properties attributed to the resulting ceramides [93]. Although there are two isoforms of DEGS, each isoform produces a different product from the same substrates. Both DEGS1 and DEGS2 catalyze the formation of the 4–5 trans double bond using redox cofactors, including NADPH and O_2_ [94,95]. Meanwhile, DEGS2 also undertakes a second step which hydrolyzes this newly-formed double bond and hydroxylates the C4 position [96]. As a result, DEGS1 produces typical ceramides, while DEGS2 produces phytoceramides.

Phytoceramides are structurally and functionally similar to the alpha-hydroxy ceramides produced by CerS3 [97]. Both possess an extra hydroxyl group located near the head of the molecule [84,97] and are potent inducers of apoptosis [85,98]. Consequently, DEGS2 expression is positively correlated with dramatically improved patient survival [39]. The DEGS2 enzyme is differentially expressed in IDH*^mut^* gliomas [39], which accounts for the presence of phytoceramides that contribute to the sphingolipid rheostat imbalance observed in the IDH*^mut^* glioma subtypes [11]. The development of pharmacological activators of DEGS2 could result in novel glioma therapies and will enhance our understanding of the signaling roles of phytoceramides.

On the other hand, DEGS1 is essential for de novo ceramide production and the formation of complex sphingolipids in the brain. This final step allows for the formation of sphingomyelin and glycosylceramides, which are essential for normal brain development and function [99]. The overexpression of DEGS1 may divert the ceramides from the sphingolipid rheostat to other metabolic fates, correlating with a lower glioma patient survival [39].

## 5. Golgi: Complex Sphingolipids Synthesis

The production of ceramide in the ER concludes the de novo portion of sphingolipid synthesis. Ceramide is the central metabolite of the sphingolipid pathway and has multiple cellular fates, such as sphingomyelin (SM), glycosphingolipids (GS), ceramide-1-phosphate (C1P), and sphingosine-1-phosphate (S1P). Each group of sphingolipids plays important role in cell growth, and all can be recycled back to ceramide via salvage pathway enzymes. Most of these metabolites, apart from S1P, are synthesized in the Golgi body. Therefore, upon their de novo production in the ER, ceramides must be transported to the Golgi body for the subsequent synthesis of sphingomyelin (SM), ceramide-1-phosphate (C1P), and glycosphingolipids (GS) to occur.

### 5.1. Ceramide Transport to the Golgi

This is typically accomplished by the ceramide transport protein (CERT), a cytosolic protein that serves as the major non-vesicular ceramide transporter in cells (Figure 2D) [100], including gliomas [101]. CERT primarily transports the pro-apoptotic C14–20 ceramides, dihydroceramides, and phytoceramides with a minimal affinity for longer-chain ceramides [102]. Notably, CERT transports ceramide for its incorporation into CNS SM [83] and C1P [103,104], but not GS [100], even though all three synthetic pathways take place in the Golgi body. Thus, the transport of ceramide for glucosylceramide (the primary GS) synthesis utilizes CERT-independent mechanisms, which require further investigation [26,105]. CERT’s lack of affinity for the C22–24 ceramides produced by CerS2 may explain why CerS2 ceramide products comprise the vast majority of glucosylceramides in the brain [83].

Studies based on probing immortalized human cells with UVB-irradiation revealed that the inactivation of CERT results in the pro-apoptotic accumulation of ceramide [26], resulting from decreased SM synthesis [26,106]. This suggests that CERT is an important regulator of the sphingolipid rheostat, as it shunts pro-apoptotic ceramides towards the Golgi for SM and C1P synthesis. However, as noted below, high sphingomyelin levels can also induce cancer cell death through unique mechanisms. Therefore, cancers likely utilize CERT to balance ceramide and SM levels and ensure minimal pro-apoptotic signaling from both metabolites. Further research is necessary to better elucidate the contribution of CERT to the sphingolipid rheostat in cancers and to assess the roles of recently identified novel inhibitors [107].

### 5.2. Production of Sphingomyelin

Upon the arrival of ceramide in the trans-Golgi network [108], sphingomyelin synthase (SGMS)1 produces SM in the Golgi lumen [109]. This enables the vesicular transport of SM to the extracellular environment (Figure 2D). One would expect the activity of SGMS to sequester cytosolic ceramide, decreasing the pro-apoptotic influence of the sphingolipid rheostat. This hypothesis holds in non-immortalized rat astrocytes, where sphingomyelin synthesis activated by bFGF signaling resulted in reduced ceramide levels and increased cell proliferation [110]. However, glioma cells have markedly reduced concentrations of SM compared to healthy cells [111]. Additional studies have revealed that excessive SM production in SF767 glioma cells can inhibit the tumorigenic EGFR/MAPK and PI3K/Akt pathways, resulting in cell death [112]. While ceramide accumulation induces apoptosis, excess SM induces differentiation, decreased proliferation, and autophagy [112]. Thus, gliomas appear to maintain homeostasis for ceramide and sphingomyelin levels that protects the cell from cumulative anti-growth signaling. SGMS1 is one of the few enzymes discussed in this review with a known activator. The drug known as 2-OHOA has been used in multiple clinical trials on brain tumors and is discussed extensively in Section 9.

### 5.3. Phosphorylation of Ceramide

Another pathway enzyme located in the trans-Golgi network is ceramide kinase (CERK) (Figure 2D) [103], which was originally identified in the brain [113]. The ceramide-1-phosphate transfer protein (CPTP) transfers C1P from the Golgi to the plasma membrane [114], where it possesses various signaling roles in several cell lines. C1P from bovine brains stimulates DNA synthesis [115], indicating that it plays a role in promoting cell proliferation. Therefore, the inhibition of ceramide kinase would likely be a useful strategy in treating gliomas with dysregulated sphingolipid metabolism.

### 5.4. Initiation of the Glycosphingolipid Synthesis Pathway

Ceramide can also be transported to the Golgi body through a vesicular mechanism that has not yet been fully elucidated [105]. Upon its arrival at the medial Golgi, ceramide is glycosylated by UDP-glucose ceramide glycosyltransferase (UGCG) (Figure 2D), resulting in a new family of sphingolipids called glycosylceramides [116]. These complex sphingolipids are metabolized into several unique glycosylated end products, which are known to promote tumorigenesis in several cancer types [117,118], including glioblastomas [119].

Due to its metabolic proximity to ceramide, UGCG is likely the glycosphingolipid synthesis enzyme that has the greatest influence on the sphingolipid rheostat. A recent study using glioblastoma cells from a patient-derived xenograft indicates that UGCG produces pro-survival glycosphingolipids [90] by consuming pro-apoptotic ceramides [11]. Notably, UGCG is differentially expressed in more aggressive cancer types, including IDH*^wt^* gliomas, where it is also correlated with worse patient survival [39]. This further validates the results of Dowdy et al., demonstrating that IDH*^mut^* gliomas possess sphingolipid vulnerabilities that result in a rheostat that favors apoptosis [11].

## 6. Plasma Membrane

Upon translocation to the plasma membrane, the sphingolipids produced within the Golgi carry out their respective signaling functions. Although a detailed discussion of their signaling activities is beyond the scope of this review, it should be noted that each sphingolipid possesses unique roles in promoting or inhibiting the hallmarks of cancer. SM, GS, and ceramides can then be recycled through the endocytic pathway (discussed in Section 7), while C1P and S1P are degraded through their respective phosphatases and lyases (discussed in Section 8).

### 6.1. Production of Sphingomyelin at the Membrane

Although both isoforms of sphingomyelin synthase (SGMS) catalyze the same reaction, their differing localizations contribute to their opposing roles in the cell. SGMS1 is most often found in the luminal leaflets of trans and medial Golgi cisternae, while SGMS2 associates with the plasma membrane [109]. Therefore, SGMS1 produces SM, which is incorporated into lipid rafts on the outer membrane leaflet that regulate the pro-tumorigenic signaling of the growth factor receptors [112,120]. Meanwhile, SGMS2, along the inner membrane leaflet, consumes cytoplasmic ceramide, decreasing the concentration of pro-apoptotic ceramides within the cell and shifting the rheostat away from apoptosis. Based on these observations, it makes sense that SGMS1 is differentially expressed in IDH*^mut^* gliomas and is correlated with improved patient survival [39]. In contrast, SGMS2 tends to be highly expressed in IDH*^wt^* gliomas and correlated with deteriorated survival [39,121]. Inhibiting SGMS2 may interfere with the ability of glioma cells to maintain ceramide and SM homeostasis, thus increasing ceramide levels and exceeding the rheostatic threshold necessary for inducing apoptosis.

### 6.2. Degradation of Sphingomyelin at the Membrane

The activity of neutral sphingomyelinase 2 (nSMase2; *SMPD3* gene) directly opposes the role of SGMS2 in the plasma membrane. nSMase2 is highly enriched in the brain [122] and plays an important role in normal neural function [123]. As an integral membrane protein [122], its lack of mobility limits its access to different pools of sphingomyelin [124]. This implies that nSMase2 exclusively degrades SM near the plasma membrane to produce ceramide. As a result, it directly counters the SM produced by SGMS2 at the membrane and encourages the rheostat towards apoptosis by promoting ceramide accumulation in the cytosol. It comes as no surprise that higher *SMPD3* expression in glioma patients dramatically improves patient survival [39]. The gene is differentially expressed in IDH*^mut^* gliomas [39], again supporting the conclusions of Dowdy et al. [11]. Therapies that can activate nSMase2 may play an important role in mitigating gliomas, particularly since its expression is concentrated in the brain.

### 6.3. Production of Sphingosine-1-Phosphate at the Membrane

Neutral ceramidase (nCDase, *ASAH2* gene) is another plasma membrane protein that plays a critical role in sphingolipid metabolism. nCDase further cleaves the ceramide products of nSMase2 to produce sphingosine [125], which possesses unique signaling functions that alter the rheostat and promote apoptosis [11] (discussed in Section 8.1). Despite its production of pro-apoptotic sphingosine, nCDase is not correlated with improved patient outcomes [39] because the sphingosine is rapidly phosphorylated by sphingosine kinase to produce S1P (discussed in Section 8.1). The minimal impact of nCDase on patient survival makes it a relatively poor target without further investigation of its role in cancer. However, its production of sphingosine makes it particularly relevant since it can directly impact the rheostat balance between ceramides and S1P. The phosphorylation of sphingosine into S1P results in the pro-survival signaling that cancers require to maintain growth.

### 6.4. Degradation of Ceramide-1-Phosphate

Ceramide 1-phosphate (C1P) phosphatase is an enzyme in the CNS [126] that hydrolyzes the phosphate head-group of C1P to release ceramide into the metabolic pool. Dephosphorylation is the only way to degrade C1P [126], which marks a notable difference from its S1P counterpart, which can also be degraded by S1P lyase. Since C1P phosphatase activity results in ceramide production, its activity likely contributes to increased ceramide levels. Therefore, its inhibition would not be a wise strategy for targeting the sphingolipid rheostat in gliomas to promote apoptosis.

## 7. Lysosomes: Salvage Pathway

As parts of the plasma membrane are endocytosed through homeostatic processes, sphingolipids from the membrane enter the salvage pathway. The endosome soon merges with acidic lysosomes containing important degradation enzymes that cleave complex sphingolipids, converting them back into ceramide and sphingosine. As seen in Figure 2E, the breakdown of SM, ceramides, and glucosylceramide in the lysosome plays an important role in recycling metabolites and provides a valuable source of targets. However, since glycosphingolipids generally support cancer growth [127], the inhibition of their degradation by glucocerebrosidase would be counterproductive to enhancing apoptosis in gliomas. Therefore, our focus in this section lies in discussing sphingomyelin and ceramide degradation.

### 7.1. Lysosomal Degradation of Sphingomyelin

Acid sphingomyelinase (aSMase) arises from the *SMPD1* gene and is typically localized in the lysosomes, depending on its post-translational modifications [128]. Most notably, aSMase can be activated in response to a long list of harmful conditions, “death receptors”, and stress pathways [129]. For example, activating the Fas/CD95 receptor results in aSMase activation and caspase cleavage in U87MG and U373 gliomas [130]. The activated aSMase then translocates to SM-rich domains on the plasma membrane, where it increases local concentrations of ceramide [131]. Both radiation and chemotherapy can selectively activate aSMase to hydrolyze sphingomyelin into pro-apoptotic ceramides [23,132]. aSMase overexpression sensitizes gliomas to gemcitabine and doxorubicin chemotherapies [132]. However, more recent studies indicated no significant differences in the effects of aSMase in combination with temozolomide-treated LNT-229 and T98G cells [133]. Additional research is necessary to evaluate the effects of aSMase overexpression on other glioma subtypes and combination therapies. As seen with the stress response pathways, activating aSMase increases ceramide levels to promote apoptosis. Given what we know about the effects of SM accumulation in the cell, inhibiting aSMase could produce apoptotic effects in gliomas as well.

### 7.2. Lysosomal Degradation of Ceramide

While ceramide has numerous potential fates, its lysosomal hydrolysis by ceramidase enzymes is a significant step in the sphingolipid pathway. This results in the formation of the anti-proliferative sphingosine, whose activity resembles ceramide-induced apoptosis in certain cancers [31]. Sphingosine can inhibit protein kinase C (PKC) and phosphatidic acid phosphohydrolase, whose activities promote oncoprotective and anti-apoptotic responses in certain cancers [134,135]. Furthermore, transformed tumorigenic and stem-like cells have demonstrated greater sensitivity to the biostatic effects of the sphingoid bases (such as sphingosine) compared to normal differentiated cells [136].

While elevated sphingosine levels contribute to a pro-apoptotic sphingolipid rheostat, certain GBM lines (e.g., U-373MG) and other malignant cancers have exhibited the overexpression of sphingosine kinase (discussed in Section 8.1) [14,18]. Sphingosine kinase activity depletes cellular sphingosine levels via phosphorylation, resulting in increased S1P production [11]. Consequently, it is anticipated that the pharmacological inhibition of ceramidase will prevent the sphingolipid bases from being shunted towards S1P production. This would shift the sphingolipid rheostat exclusively towards the accumulation of ceramide while diminishing the pools of sphingosine essential for S1P production.

Figure 2E demonstrates how the location of acid ceramidase (ASAH1) in the lysosome places it in the best position to produce sphingosine for S1P production. ASAH1 is also directly associated with radioresistance, a poor prognosis, and upregulation in primary GBM tumors [30,39] and other cancers. Meanwhile, neutral ceramidase isoforms (i.e., ASAH2/ASAH2B) are associated with a better prognosis and are elevated in IDH*^mut^* glioma subtypes [39]. The expression of alkaline ceramidase (ACER) isoforms shows no correlation with patient survival [39,137]. Therefore, ceramidase-related interventions should selectively target lysosomal acid ceramidase (ASAH1).

## 8. Cytoplasm: Exiting the Sphingolipid Pathway

The release of sphingosine from salvage pathway lysosomes places it near a seminal point of the sphingolipid pathway. At this stage, sphingosine can either be recycled back into the pathway and consumed by (dihydro)ceramide synthase at the ER membrane, or it can be phosphorylated by sphingosine kinase (SPHK). S1P can then induce numerous pro-survival signaling pathways, before being degraded by either sphingosine-1-phosphate phosphatase or sphingosine-1-phosphate lyase.

### 8.1. Phosphorylation of Sphingosine

The sphingosine produced in the lysosomes by ASAH1 can be phosphorylated by sphingosine kinase (SPHK) to produce S1P. While both SPHK1 and SPHK2 participate in the phosphorylation of sphingosine, the expression of SPHK1 is elevated in IDH*^wt^* glioma patients and is highly correlated with a worse prognosis compared to SPHK2 [39]. S1P has been classified as an oncopotent second messenger, which acts as a G protein-coupled receptor (GPCR) ligand to mediate intracellular signaling [138]. Additionally, S1P utilizes multiple targets to stimulate complex cellular processes (chemotactic motility, migration, differentiation, proliferation, apoptotic survival response, capillary permeability, differentiation, and angiogenesis) involving the key hallmarks of cancers [138]. Notably, endogenous analogs of S1P (phytosphingosine-1-phosphate, sphinganine 1-phosphate, and ceramide 1-phosphate) are bio-effectors that also modulate the global pro-survival responses across tumor subtypes [139]. S1P and ceramide maintain a negative correlation via the sphingolipid rheostat balance, in which the upregulation of ceramide is directly correlated with the concomitant downregulation of S1P, and vice versa [31].

### 8.2. Dephosphorylation of Sphingosine-1-Phosphate

Sphingosine-1-phosphate phosphatase (SGPP) is one of two possible means of decreasing S1P levels. SGPP achieves this by hydrolyzing the phosphate group from S1P to produce sphingosine, which supports the pro-apoptotic signaling of ceramide [11]. Although much of the sphingosine may be rephosphorylated, the activity of SGPP can result in short-term reductions in S1P levels. SGPP is located at the ER [140]; thus, the ER-localized SK2 produces S1P that is quickly dephosphorylated and reincorporated into the ceramides through the activity of the ceramide synthase [141,142], which is also located in the ER. Therefore, SGPP inhibition will likely not be a viable strategy for treating glioma patients.

### 8.3. Degradation of Sphingosine

Sphingosine-1-phosphate lyase (SGPL) is the final enzyme in the pathway and the only means of degrading the long-chain bases that are dedicated to sphingolipid metabolism. It cleaves S1P into phosphoethanolamine and hexadecenal, which are then recycled in the cell and assigned to any number of metabolic fates. The ablation of S1P lyase in mouse brains resulted in the accumulation of S1P [143], altering the sphingolipid rheostat of gliomas towards proliferation [11]. This unfavorable outcome indicates that S1P lyase inhibition is not a viable treatment option for gliomas. On the contrary, its activity reduces cellular S1P levels, and can also support the generation of ceramides in response to stressful stimuli [144]. Molecular activators of S1P lyase will likely be useful pharmacological tools, but none have been discovered to date.

## 9. Current Advancements in Sphingolipid Targeting

Glioma research has undergone significant growth over the past several years, as recent biochemical discoveries are translated into novel therapies. Despite this progress, many of these therapies remain in preclinical models, while the median survival of patients suffering from glioblastoma (GBM) remains well under one year [3]. This demonstrates the urgent need for continued research on therapies. In this section, we seek to highlight which drugs have successfully made it to clinical trials, and which preclinical drugs can manipulate the glioma rheostat and serve as future clinical agents.

**Figure 3:**
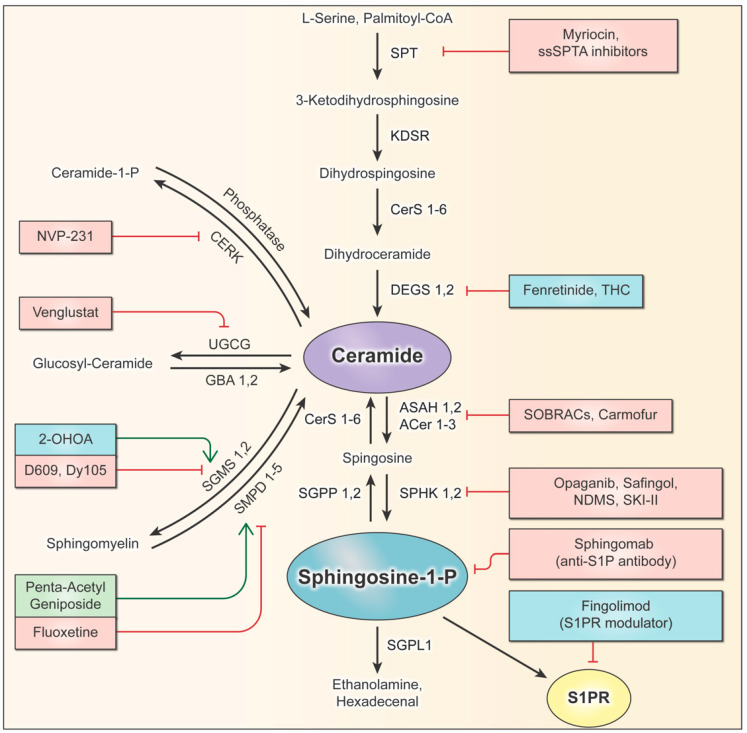
Sphingolipid pathway and the fates of ceramide. Drugs that have been involved in clinical trials on glioma patients (listed in Table 1) are in blue boxes. Pre-clinical drugs are listed in red or green boxes, depending on their status as inhibitors or activators. Additional research translating preclinical drugs may result in novel clinical trials on glioma patients and other cancer types. SPT, serine palmitoyl-CoA transferase; KDSR, keto-dihydrosphingosine reductase; CerS, ceramide synthase; DEGS, dihydroceramide desaturase; SGMS, sphingomyelin synthase; SMPD, sphingomyelinase; CERK, ceramide kinase; C1PP, ceramide 1-phosphate phosphatase; UGCG, UDP-glucose ceramide glucosyltransferase; GBA, glucocerebrosidase; ASAH/ACER, ceramidase; SPHK, sphingosine kinase; SGPP, sphingosine phosphate phosphatase; SGPL, sphingosine phosphate lyase.

The sphingolipid pathway and rheostat, balancing pro-apoptotic ceramides and pro-survival S1P, have long been postulated to be key regulatory factors that determine cell growth. Since cancers such as gliomas manipulate the sphingolipid rheostat to promote their survival, the pathway provides an abundance of potential targets that can be exploited to limit the growth of cancer. Each pathway enzyme is illustrated in Figure 3, which summarizes the sphingolipid pathway in a stepwise process and highlights the pre-clinical and clinical drugs discussed in this review. To date, four drugs have successfully entered clinical trials for gliomas (Table 1), thus forming the start of a promising avenue for drug development focused on sphingolipid modulators.

### 9.1. Drugs Targeting the Sphingolipid Pathway Assessed in Glioma Clinical Trials

Fenretinide was investigated as an anti-cancer therapy before its effects on sphingolipid production were elucidated. Its mechanism of action has since implicated a wide variety of cellular pathways, including ROS production [145], which may limit the redox status of the cell and indirectly inhibit dihydroceramide desaturase (DEGS) activity (discussed in Section 4.3) [94]. Fenretinide’s effective induction of glioma apoptosis in vitro in multiple high-grade glioma cell lines [146,147] and additional testing led to a phase-II clinical trial on patients with recurring glioma and GBM [148]. However, the trial concluded that fenretinide was ineffective against gliomas at the administered concentrations (Table 1). Additional research on improved dosing may increase the effectiveness of fenretinide in future trials.

Furthermore, Δ9-tetrahydrocannabinol (THC), an active cannabinoid in *Cannabis sativa* L. plants, has also garnered much attention for its inhibition of DEGS1 in gliomas (Table 1) [149,150]. Preclinical studies and a follow-on pilot clinical study involving THC also revealed its biostatic effects against recurrent GBM, while sparing non-transformed astroglial cells [151]. Therefore, the further development of cannabinoid (e.g., THC) analogs that demonstrate selective antitumor responses could sensitize the GBM and glioma subtypes to an imbalance in the sphingolipid rheostat. Recent clinical trials testing various drug combinations (Table 1) have demonstrated the safety of cannabinoids when used to reduce the emetic symptoms associated with harsh chemotherapy regimens in patients. However, further research is required to quantify the in vivo effects of THC on sphingolipid metabolism and glioma apoptosis.

Another drug, known as fingolimod (FTY720), was designed to improve the biostatic properties of the serine palmitoyltransferase (SPT) inhibitor called myriocin. However, this synthetic sphingolipid analog is also a competitive substrate of sphingosine kinase (discussed in Section 8.1) [152]. The phosphorylated fingolimod-1-phosphate then modulates S1P receptor 1 (S1PR1) in the CNS lymph nodes, making it a potent immunosuppressant and a successful treatment for multiple sclerosis [153]. A clinical trial investigating fingolimod in the relief of lymphopenia symptoms in high-grade glioma patients was completed in 2017. However, the results have not yet been published (Table 1). Fingolimod’s role as an SPT inhibitor and competitive inhibitor of sphingosine kinase is likely overshadowed by its modulation of the S1P receptor activity. This scenario is reflected in the recent clinical trial, whose primary focus was on examining lymph-related symptoms as opposed to sphingolipid metabolism and glioma growth.

The progress made in the investigation of these three drugs pales in comparison to that of 2-hydroxyoleic acid (2-OHOA), which is a highly specific and effective activator of sphingomyelin synthase 1 (SGMS1). It has been tested extensively in glioma cell lines, preclinical animal studies [111,112,121,154], and a phase-I/IIA clinical trial on patients. which demonstrated its safety and efficacy in humans (Table 1). One in vivo xenograft mouse study indicated that 2-OHOA may be even more effective and result in fewer tumor relapses than TMZ, the current standard of care for glioma patients [112]. Although the EC_50_ for 2-OHOA is relatively high, its oral bioavailability, low toxicity, and minimal side effects present few risks when administered at therapeutic doses. At present, phase-IIB/III trials that combine 2-OHOA, TMZ, and radiotherapy are ongoing. If the trial proceeds as expected, the European Medicines Agency has indicated that 2-OHOA could become conditionally available to glioma patients in Europe. This is the closest that a novel sphingolipid modulator has come to being approved as a therapy for gliomas.

### 9.2. Future Drug Development

Drugs such as 2-OHOA can serve as models for identifying new pathways of therapeutic research on gliomas and other cancers. The specific targeting of the SGMS1 isoform by 2-OHOA makes it particularly effective at distinguishing between pools of sphingolipids, which have different functions depending on their localization and composition. The sphingolipid pathway is full of enzymes, receptors, proteins, and other potential targets that can be exploited for therapeutic development. Many enzymes throughout the pathway are modulated by promising drugs that simply have not progressed beyond pre-clinical testing, which are also listed in Figure 3. Some of these drugs have been tested clinically for other diseases and can potentially be repurposed for use in cancer therapies. Other pathway enzymes and supporting proteins play important roles in the glioma sphingolipid rheostat and should be investigated further for novel drug development.

### 9.3. Pre-Clinical Drugs Targeting De Novo Sphingolipid Synthesis

Drug discovery efforts to target enzymes in the de novo pathway have focused on two enzymes, SPT and DEGS, resulting in several preclinical inhibitors. As discussed previously, inhibiting de novo synthesis enzymes can reduce the total sphingolipid synthesis, but this may not have a significant impact on the sphingolipid rheostat. This potential limitation has not deterred scientists and physicians from developing and testing de novo sphingolipid inhibitors in gliomas, resulting in clinical trials (Table 1) and preclinical studies.

Myriocin, also known as ISP-1 or thermozymocidin, is the most significant xenobiotic in this family of SPT-specific inhibitors. Myriocin structurally resembles sphingosine and has been investigated thoroughly due to its potency and specificity for SPT. Notably, myriocin can suppress the proliferation of multiple cancer types by altering the sphingolipid rheostat [74,155]. When tested in U87MG IDH*^wt^* glioblastoma cells, only 1 μM of myriocin was required to elicit an over 50% reduction in the cell count, following a five-day incubation period [74]. This resulted in statistically significant alterations in the sphingolipid concentrations and decreased the S1P levels by 35% [74].

Some IDH*^wt^* glioblastoma cell lines (e.g., the tumorigenic U87MG GBM line) are well-known for shunting ceramide towards S1P, which promotes tumor cell survival [13]. Therefore, the de novo synthesis inhibition of U87MG cells was shown to interrupt S1P production, resulting in apoptosis [74]. On the other hand, IDH*^mut^* cell lines favor ceramide production over S1P [11]; thus, inhibiting de novo synthesis in these glioma lines is unlikely to produce meaningful effects. Therefore, SPT and other de novo synthesis enzymes serve as better targets in the case of higher-grade gliomas. Two recent brain tumor clinical trials (discussed above) involving therapies that inhibit de novo synthesis enzymes specifically recruited patients with higher-grade gliomas or glioblastoma multiforme.

In addition to the competitive inhibition by myriocin, as observed above, the application of high-throughput screening and medicinal chemistry has facilitated the discovery of other structurally diverse SPT inhibitors with unique mechanisms of action. This novel and structurally diverse drug class bind directly to the ssSPTa subunit (rather than targeting the catalytic subunits), thus preventing full SPT activity [156]. The antitumoral activities of these SPT inhibitors have been demonstrated in acute myeloid leukemia (AML) xenograft mouse models [157] and lung adenocarcinoma cells [158] and can be readily applied to gliomas. Given the evidence suggesting that de novo biosynthesis can be targeted to disrupt the sphingolipid rheostat of certain IDH*^wt^* gliomas [74], this novel class of SPT inhibitors should be evaluated as potential therapeutic strategies against heterogeneous glioma lines [155,157,158].

The most well-known inhibitor of DEGS is the vitamin analog fenretinide (4-HPR), whose anti-cancer properties have been studied extensively, resulting in the clinical trial discussed in Table 1. Other clinically relevant DEGS inhibitors include THC, also discussed previously. Administering THC to glioma cells alters the dhCer-to-ceramides ratio, resulting in autophagy followed by cell death in vitro [150]. Similar to ceramides, the related stimulation of cannabinoid-specific G protein-coupled receptors by cannabinoids (e.g., THC and endocannabinoid 2-arachidonoylglycerol, 2-AG) mediates ER stress through the activation of stress-induced p8 expression in certain cancers, including GBM, in vitro [151,159].

Another drug known as SKI-II [160] has been proven to effectively inhibit sphingosine kinase (SPHK)1 and SPHK2 (discussed in Section 8.1). Interestingly, SKI-II can also noncompetitively inhibit the activity of the DEGS1 [161]. It is suspected that SKI-II also mediates the inhibition of DEGS, affecting the cellular redox status. In T98 GBM cells, the in vitro use of SKI-II resulted in the accumulation of complex dihydroceramides (dhCers) through its inhibition of DEGS1, as well as the near depletion of S1P due to its primary inhibition of SPHK [161]. Upon testing SKI-II as a maintenance therapy alongside temozolomide (TMZ), SKI-II affected TMZ cytotoxicity, which induced cell stress, autophagy, and apoptosis in several GBM cell lines, including the TMZ-resistant cell lines [29].

### 9.4. Pre-Clinical Drugs Targeting Complex Sphingolipid Synthesis

In addition to SGMS1, the Golgi hosts two other important targets that are responsible for the synthesis of pro-survival complex sphingolipids. Both ceramide kinase and UGCG are present in different regions of the Golgi and possess known inhibitors that have been minimally tested in gliomas. Therefore, these established drugs can be repurposed for testing in brain tumors.

NVP-231 is a potent and reversible inhibitor of ceramide phosphorylation [162]. It induces the arrest of the cell cycle and inhibits the proliferation of both breast and lung cancer cells by decreasing C16-C1P levels and increasing the total ceramide levels [163]. Notably, it seems to mediate cell death by inducing cell cycle arrest. Additional studies on breast cancer indicated that treating cells with NVP-231 also resulted in reduced migration and invasion [164]. Given these promising studies, CERK inhibitors such as NVP-231 should be examined in glioma cells to determine their capacity for manipulating the sphingolipid rheostat and inducing apoptosis.

The therapy Genz-682452 (venglustat) is currently the most widely studied UGCG inhibitor because of its relative safety, potency, and oral bioavailability [165]. It has been used in several clinical trials on diseases that involve the dysregulation of the glycosphingolipid biology, such as kidney disease, Fabry disease, Gaucher’s disease, and others. This research is strengthened by animal model studies, which confirmed that venglustat is a strong CNS penetrant that can decrease glycosphingolipid levels in the brain by more than 20% [166,167]. Based on these results, venglustat appears to be a viable candidate for testing in glioma patients or patients with other cancers with aberrant sphingolipid metabolism. To date, no clinical trials have been initiated to illuminate venglustat’s role in cancer, and minimal research has been conducted to assess its effects on cancer biology. The structurally similar GZ667161 is another CNS-penetrant UGCG inhibitor, which has been studied in mouse models of Parkinson’s disease, and it has also been shown to reduce brain glycosphingolipid levels [168]. These results also warrant future investigation to elucidate the role of GZ667161 as an anti-cancer agent.

Other UGCG inhibitors include the glucosylceramide analog PDMP (1-phenyl-1-decanoylamino-3-morpholino-1-propanol). Notably, D-PDMP was shown to penetrate the blood-brain barrier and decrease the tumor volume of C6 glioma cells in rat models, with minimal behavioral side effects [169]. The D-PDMP treatment of C6 glioma cells was also found to augment ceramide levels, particularly upon etoposide treatment, which resulted in caspase activation [170]. This treatment has also demonstrated promising results in mouse models of renal cancer, decreasing the tumor volume and modulating the levels of various sphingolipids [171]. However, some effects of D-PDMP are not attributed to its UGCG inhibition, and additional experimentation is required to elucidate the other mechanisms of action [172]. Other novel brain-penetrant drugs, such as T-036, have recently been discovered and require additional investigation to determine their capacity for inhibiting cancer growth [173].

### 9.5. Pre-Clinical Drugs Targeting Sphingolipid Metabolism at the Membrane

D609 is a putative SGMS inhibitor [110], but its mechanism of action is broad and complicated [174]. A study examining the D609 inhibition of glioma growth did not even consider its effects on SM levels or collect any metabolic data regarding the sphingolipids [175]. Notably, D609 achieves its effects by interfering with EGFR and AKT signaling in the U87MG cells, similar to the mechanism of action observed with the 2-OHOA [112]. Specific SGMS inhibitors have also been developed and optimized, such as the D2 group of drugs [176]. For example, Dy105 preferentially inhibits the tumorigenic SGMS2, with an IC50 value below 20 μM [149]. However, the acetonitrile functional group on the molecule is expected to pose threats of toxicity in animal and human trials.

The compound penta-acetyl geniposide has been found to activate nSMase2 and inhibit the growth of C6 glioma cells [177], as well as tumors in in vivo rat studies [178,179]. However, as with most non-specific drugs, penta-acetyl geniposide performs multiple actions in gliomas, and its effects are not entirely attributed to the nSMase2 activation [180]. Further studies must be conducted to determine whether penta-acetyl geniposide is a viable drug for additional in vivo testing.

### 9.6. Pre-Clinical Drugs Targeting Lysosomal Sphingolipid Degradation

Due to the significance of the lysosomal enzymes to the glioma rheostat, great attention has been paid to the investigation of novel therapies that target acid sphingomyelinase and acid ceramidase. In addition, the uniquely acidic environment of the lysosomes can be exploited as a tool for the specific delivery or activation of drugs designed to target lysosomal enzymes. These insights guide current and future drug discovery efforts in making potent and specific therapies for glioma patients.

Recent studies indicate that the selective serotonin reuptake inhibitor (SSRI) fluoxetine can inhibit aSMase and induce glioma cell death [120]. The excess accumulation of SM suppresses the EGF receptors [120], which are known to activate sphingosine kinase and subsequent S1P production [181]. Bi et al. also examined retrospective SSRI studies [182,183], extracted data relating to fluoxetine, and determined that patients taking fluoxetine did indeed experience improved survival.

Given that the safety and BBB penetration of fluoxetine are already well-established, the next steps in this research will likely involve an experimental clinical trial on glioma patients. It is unclear how the inhibition of aSMase by fluoxetine competes with the concomitant enzyme activation by chemotherapy, or how this affects its dosing. Therefore, the optimal anti-cancer dosing should be investigated in future trials, as it likely differs from the proper antidepressant dosing.

Recently developed ASAH1 inhibitors (e.g., N-oleoylethanolamine, B13, and benzoxazolone carboxamides) have been defined as chemosensitizers that can be combined with conventional chemo- and radiotherapies against human glioma and other (prostate and L929) tumor cells in vitro, as well as in xenograft models [137,184]. Other ASAH1 inhibitors, such as carmofur, reduce S1P production and possess potent biostatic effects that sensitize GBM, adenocarcinoma, and hepatoma cells to chemotherapy [25,30].

Carmofur is the pro-drug of the antineoplastic agent 5-fluorouracil (5-FU), which has been used extensively in the treatment of breast and colorectal cancer [185]. Its lipophilic structure allows it to be administered orally before being converted into 5-FU upon absorption [186]. Notably, it easily crosses the blood-brain barrier and is a potent inhibitor of ASAH1 [185], making it a worthy drug for investigation in the case of gliomas. To date, it has been shown to slow the growth of temozolomide-resistant GBM cells [187], as well as human prostate and breast cancer cell lines [188], among other cancer types.

Another group of potent inhibitors is comprised of the haloacetate ceramide derivatives arising from the compound sphinganine-2-bromoacetate (SABRAC). SABRAC and its related compounds have shown positive results in prostate cancer cells [189] and in vivo acute myeloid leukemia mouse models [190]. However, unlike carmofur, their degrees of bioavailability and blood-brain barrier penetration is still unknown. Further investigation is required to assess whether this family of drugs can serve as therapies for glioma patients.

### 9.7. Pre-Clinical Drugs Targeting Sphingosine Kinase

Sphingosine kinase has been shown by several studies as one of the key oncogenic enzymes in the sphingolipid pathway [11,13,191] and has been the focus of great attention. A recent study by Dowdy et al. demonstrated a novel approach to arresting the proliferation and causing the apoptosis of IDH*^mut^* glioma subtypes (TS603, BT142, and NCH1681), specifically by modulating the sphingolipid rheostat [11]. This approach utilized the SPHK1 inhibitor (N,N-dimethylsphingosine, NDMS) to restrict S1P production, in combination with the addition of C17 sphingosine to further promote a metabolic shift toward the pro-apoptotic accumulation of ceramide and sphingosine. This study discovered a metabolic vulnerability involving the global elevation of ceramides, in conjunction with suppressed SPHK2 expression in the representative IDH*^mut^* gliomas compared to IDH*^wt^*. The findings revealed that metabolic vulnerabilities involving the sphingolipid rheostat can be targeted to produce potent anti-prolific and pro-apoptotic treatments against IDH*^mut^* gliomas, specifically. In a high-throughput screening with commercially available SPHK inhibitors (i.e., ABC294640, mp-A08, PF-543, and SLP7111228) and the S1P receptor (S1PR) antagonist (VPC 23019), NMDS was established as the most potent inhibitor against representative IDH*^mut^* gliomas [11].

The orally available SPHK2 inhibitor ABC294640 has participated in phase-II clinical trials against cholangiocarcinoma and related solid tumors [192,193]. In comparison, one potent SPHK inhibitor PF-543 was not considered an effective anticancer treatment [193]. However, confocal microscopy using a boron–dipyrromethene (BODIPY) PF-543 fluoroprobe established this inhibitor’s primary localization in the cytosol of A549 cancer cells, which can validate its usage in biological studies [193]. Although NDMS has not been widely researched due to its low aqueous solubility, the ongoing study that demonstrated the selective suppression of IDH1*^mut^* over IDH1*^wt^* gliomas is pending the development of xenograft models [11,193].

Another novel approach currently being undertaken is the development of a novel anti-S1P monoclonal antibody that has shown potential as a novel therapeutic [194]. It tags the S1P molecules in the tumor microenvironment, neutralizing their ability to signal for the hallmarks of cancer, such as growth, invasion, and angiogenesis. A phase-II clinical trial completed in 2017 attests to this antibody’s relative safety for use in renal cell carcinoma patients and encouraged future investigation to better determine its efficacy in other cancer types (NCT01762033). Due to the role of S1P in voracious glioma growth, sonepcizumab may be a useful treatment option for future patients.

## 10. Perspectives and Concluding Remarks

Throughout this review, we have analyzed the core enzymes of the sphingolipid pathway and assessed their capacity for acting as novel targets in cancer therapies. Although the field of cancer biology has made many exciting discoveries recently, these have, thus far, not been translated into viable therapies for many cancers, including gliomas. We hope that this review provides insight into the targets and existing therapies of the sphingolipid pathway, a recently discovered vulnerability in certain glioma subtypes. This may provide insight into the future development of novel drugs or the repurposing of existing drugs to improve glioma patient outcomes and quality of life.

**Figure 4:**
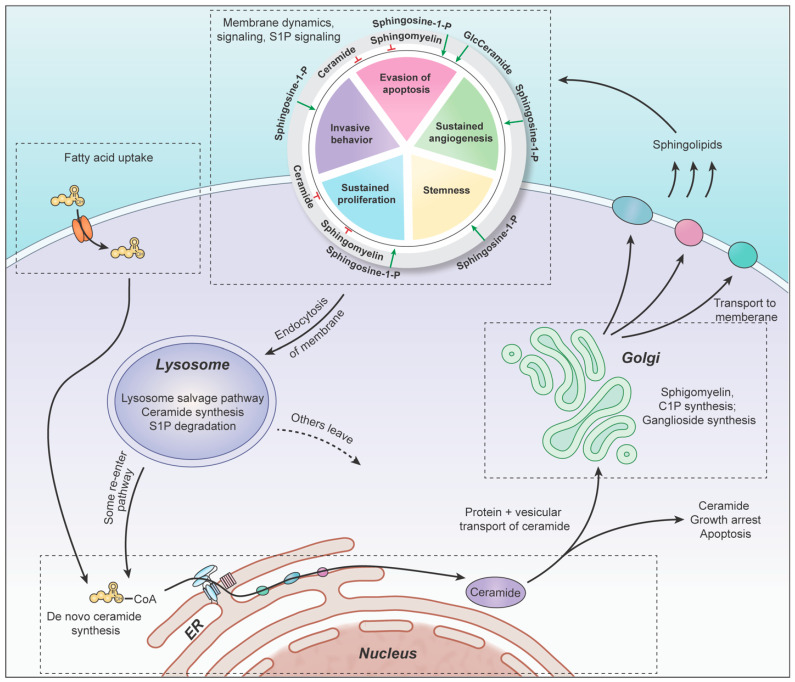
Compartmentalization of the various parts of sphingolipid metabolism. Each compartment is marked by a dashed rectangle. Fatty acids such as PA are taken up by the cell and transported to the ER, where they are incorporated into de novo ceramide synthesis. The ceramide is then transported to the Golgi for the synthesis of complex ceramides, such as sphingomyelin or gangliosides, which are sent to the plasma membrane to carry out their signaling functions, impacting the hallmarks of cancer. As parts of the membrane are endocytosed, sphingolipid metabolites are degraded by the lysosomal enzymes and recycled. The levels of these metabolites affect different hallmarks of gliomas.

There are several complexities involved in targeting the sphingolipid biology that must be taken into consideration. Given that ceramide species have specific metabolic destinations based on the length and unsaturation of their fatty acid moieties, salvage pathway enzymes produce different types of ceramides in different regions of the cell. For example, sphingomyelin is produced in the trans-Golgi loci using C14–C20 ceramide, while glycosylceramides are typically produced in the cis-Golgi loci and utilize the C22–C24 ceramides. Salvage pathway hydrolysis of the sphingomyelins and glycosylceramides generate different species of ceramides in different cellular compartments. Therefore, ceramides may be unique in composition and localization, depending on their metabolic origin, which impacts their bioactivity. As a result, it is critical to understand where each metabolic step takes place, and how metabolites are transported from one organelle to another. Figure 4 summarizes our location-specific discussion and provides an overarching view of the whole sphingolipid pathway throughout the cell.

Numerous studies have proposed different or opposing cellular roles for specific sphingolipids. One likely explanation for these contradictions is that many of the experimental techniques used to investigate sphingolipids do not preserve information regarding the localizations and fatty acid compositions of the lipid species in question. This challenge, and the related lack of standardization, can account for the misleading and contradictory findings, resulting in uncertainty that hinders the translation of therapies from drug discovery to clinical treatment. Advanced lipidomic testing through single-organelle spectroscopy can provide more accurate and detailed information about lipid localization and constitution. Additionally, utilizing tools such as Raman microscopy [195] and fluorescence [196], as well as liquid chromatography/mass spectrometry [11], can shed light on the localization, composition, and roles of specific species.

Another subtlety to consider is that enzyme isoforms can assign sphingolipids to specific metabolic destinations. While current metabolic targeting does not discriminate between the isoforms of an enzyme, inhibiting individual isoforms in the sphingolipid pathway may produce the desired effects. This is particularly evident in the specific activation of SGMS1 by 2-OHOA. A general sphingomyelin synthase activator would also impact the oncopotent SGMS2, which would be counterproductive as a glioma therapy. However, the specific activation of SGMS1 has resulted in a promising potential drug that is currently in phase-III clinical trials. This nuanced view of sphingolipid metabolism in cancer requires a step-by-step analysis to examine each enzyme in the pathway. Therefore, we approached this review by discussing the potential benefits of inhibiting individual isoforms and highlighting the key drugs and targets that could alter the sphingolipid rheostat in gliomas to induce apoptosis.

Despite these potential obstacles, the sphingolipid rheostat is a promising area of cancer biology that has great potential for translation into clinical findings. This process has already begun, as the number of preclinical and clinical studies involving sphingolipid modulators continues to increase. Over the coming years, these additional studies will fill important gaps in knowledge. Coupled with an improved understanding of the mechanisms that control the sphingolipid rheostat, this research will help to bridge the gap to improve future anti-cancer therapies.

## Figures and Tables

**Table 1:** Clinical trials on sphingolipid modulators in brain tumor.

Drug	Target	Study Type	Status	Tumor Type
Fenretinide	DEGS1 inhibitor	Phase II (NCT00006080)	Fenretinide was inactive against recurrent glioma at the dosage used	Malignant glioma
Fenretinide	DEGS1 inhibitor	Phase I NCT00003191)	Data support phase-II trial in neuroblastoma	Neuroblastoma
Fenretinide lipid matrix	DEGS1 inhibitor	Phase I (NCT00295919)	Phase-II trial recommended	Neuroblastoma
Cannabis and Temozolomide	DEGS1 inhibitor	Phase I (NCT03246113)	Terminated	Glioblastoma
THC + CBD combo and Temozolomide	DEGS1 inhibitor	Phase I–II (NCT03529448)	Ongoing	Glioblastoma
Sativex and Temozolomide	DEGS1 inhibitor	Phase I–II (NCT01812616)	Acceptable safety and tolerability, survival differences observed	Glioblastoma
Dronabinol	DEGS1 inhibitor	Phase I (NCT00314808)	Potential use as anti-emetic to improve quality of life	Primary Gliomas
2-OHOA and Temozolomide	SGMS1 activator	Phase I (NCT03867123)	Results not publicly available yet	Glioblastoma
2-OHOA	SGMS1 activator	Phase I–II (NCT01792310)	2-OHOA is well tolerated, antitumor activity warrants further trials	Gliomas and other solid tumors
2-OHOA and Temozolomide	SGMS1 activator	Phase II-III (NCT04250922)	Ongoing	Glioblastoma
2-OHOA	SGMS1 activator	Phase I–II (NCT04299191)	Ongoing	High grade glioma
Fingolimod	S1PR modulator	Phase I (NCT02490930)	Results not publicly available	High grade glioma

## Data Availability

Not applicable.
